# Evaluating Use Patterns of a Closed Electronic Nicotine Delivery System Among Adults in the United States Who Smoke Cigarettes Daily: 8-Week Actual Use Study

**DOI:** 10.2196/76019

**Published:** 2025-08-25

**Authors:** Steve Roulet, Claudia Kanitscheider, Pierpaolo Magnani, Gerd Kallischnigg

**Affiliations:** 1 Philip Morris Products S.A., Quai Jeanrenaud 5, Neuchâtel, 2000, Switzerland, 41 582425338; 2 Oracle Life Sciences, Oracle Corporation, Austin, TX, United States; 3 ARGUS GmbH, Berlin, Germany

**Keywords:** e-cigarettes, electronic nicotine delivery systems, tobacco use, use patterns, smoking reduction, quitting smoking

## Abstract

**Background:**

Adults who smoke cigarettes may reduce their exposure to cigarette smoke by switching completely or partially to an electronic nicotine delivery system (ENDS).

**Objective:**

This study investigated the use of a novel ENDS over 8 weeks, under near real-world conditions, among adults who smoke cigarettes daily and are living in the United States. The objectives were to assess the proportion of participants who switched completely to the novel ENDS and changes in cigarette consumption.

**Methods:**

This actual use study assessed the use of the novel ENDS among exclusive cigarette smokers (n=353) and dual users of cigarettes and ENDS (n=367). Participants were required to be aged 21 to 64 years old, had smoked 100+ cigarettes in their lifetime, smoked cigarettes daily at the time of enrollment, and reported positive intention to try, use, and buy the study product (SP). Participants were recruited from consumer databases. During the baseline period, participants self-reported their daily use of cigarettes and ENDS using an electronic diary. During the observational period, self-reporting also included daily use of SP. The SP was available in 2 variants (tobacco and menthol) at one nicotine concentration (3.5%; 39 mg/mL nicotine). Participants obtained 10 cartridges at the beginning of the observational period and could select either SP variant or both and were allowed to obtain additional SP. SP use patterns and sensory experiences were assessed at the end of the observational period, and cigarette consumption was compared to baseline. SP variant use and its impact on complete switching and cigarette consumption were also assessed.

**Results:**

The average age of enrolled exclusive cigarette smokers and dual users was 47 (SD 10.4) years and 44.3 (SD 9.6) years, respectively, and there were more female than male participants (227/353, 64.3% and 186/367, 50.7%). Participants smoked on average 13.3 (SD 7.3) and 12.1 (SD 7.2) cigarettes per day, and mostly menthol cigarettes (197/353, 55.8% and 201/367, 54.8%). Most participants had no plan to quit cigarette smoking in the next 6 months (261/353, 73.9% and 328/367, 89.4%). At the end of the observational period, 86.2% (304/353) of exclusive cigarette smokers and 92.1% (338/367) of dual users used the SP; 4% (14/353) of exclusive cigarette smokers and 4.1% (15/367) of dual users switched completely to the SP. Furthermore, 25.8% (91/353) of exclusive cigarette smokers and 28.1% (103/367) of dual users substantially reduced their cigarette consumption (50% or more) compared to baseline. Finally, around half of the participants (exclusive cigarette smokers: 166/304, 54.6%; dual users: 167/338, 49.4%) used only the menthol SP variant, which was also more positively evaluated in taste, smell, and aftertaste than the tobacco SP variant.

**Conclusions:**

This study indicates that adults who smoke cigarettes can switch completely to the novel ENDS or substantially reduce their cigarette consumption. These results complement the scientific evidence suggesting that ENDS are an acceptable alternative to cigarettes for adults who smoke in the United States.

## Introduction

Nearly 36 million adults in the United States smoked cigarettes or other combusted tobacco products in 2021 [1]. Exposure to cigarette smoke is harmful to both individual and public health, increasing the risks of developing many serious diseases [2-5].

The adverse health impacts of smoking can be mitigated by preventing smoking initiation and promoting smoking cessation among adults who smoke cigarettes. Such adverse health impacts can also be reduced among adults who would otherwise continue to smoke by transitioning them away from cigarettes toward alternative tobacco and nicotine-containing products (TNP) that present lower risks to health, such as electronic nicotine delivery systems (ENDS) [6].

Estimates from population health modeling suggest that complete switching from cigarettes to ENDS among adults who smoke cigarettes in the United States could prevent approximately 6.6 million tobacco-related deaths over a 10-year period [7]. Switching to ENDS is the most common method used by adults who smoke cigarettes in the United States when attempting to quit smoking [8-10]. Findings from cohort studies and randomized controlled trials summarized in a recent Cochrane Review demonstrate higher rates of smoking reduction and quitting among adults who smoke cigarettes and use ENDS than those who use other methods, such as nicotine replacement therapies (NRTs). Adults who smoke cigarettes and who completely or partially switch to ENDS are shown to smoke significantly fewer cigarettes per day (CPD) and have increased smoking abstinence compared to those using NRT, behavioral support, and no intervention [11].

Adults who smoke cigarettes, who switch completely or partially from cigarettes to ENDS and thus substantially reduce cigarette consumption—often defined as >50% fewer CPD [12,13]—may reduce their exposure to the harmful and potentially harmful constituents present in tobacco smoke [14,15]. In addition, a substantial reduction in cigarette consumption is associated with making more quit attempts [16,17], reduced cigarette dependence [18-20], and increased likelihood of future smoking cessation [16,17,21-26]. Thus, among adults who would otherwise continue to smoke, switching completely or partially from cigarette smoking to ENDS use may reduce the adverse health impacts of smoking, given the known benefits of smoking cessation or substantial reductions in cigarette consumption [27-32].

The aim of this actual use study was to investigate use patterns of a novel closed-system ENDS (the study product [SP]), available with tobacco and menthol e-liquid prefilled cartridges, and changes in cigarette consumption among adults who smoke cigarettes daily in near real-world conditions over an 8-week period in the United States. The study objectives were to assess (1) the proportion of participants who switched completely to the SP, (2) changes in cigarette consumption, and (3) study objectives 1 and 2 stratified by the SP flavor variants used.

## Methods

### Study Design

**Figure 1.**
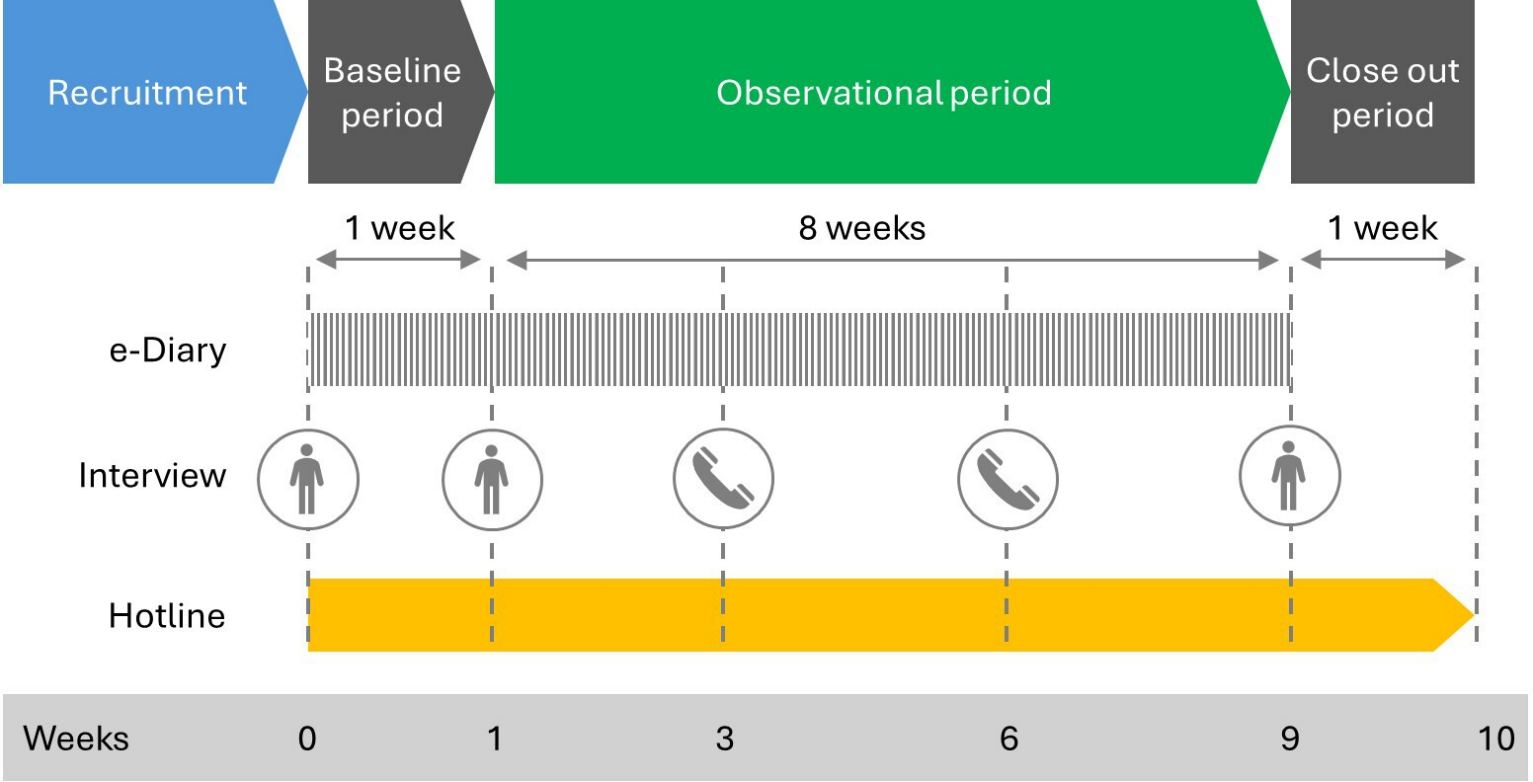
Study design.

This actual use study was conducted from May 24, 2022 (first enrollment) to October 3, 2022 (last completion) among adults who smoke cigarettes daily in the United States, including exclusive cigarette smokers and dual users of cigarettes and ENDS (hereafter, dual users). Participation in the study consisted of 3 phases: (1) a 1-week baseline phase to assess cigarette and other TNP use prior to the introduction of the SP; (2) an 8-week observational phase where participants were able to consume the provided SP and other TNP ad libitum under near real-world conditions, allowing assessment of SP use and changes in cigarette and other TNP use; and (3) a 1-week closeout phase for continued surveillance of potential adverse events. Figure 1 presents the phases of the study design. The study design was implemented following similar actual use studies [33-35].

### Participants

Participants were recruited from consumer databases of 7 geographically distributed study sites in the United States (Atlanta, Baltimore, Miami, Nashville, Orlando, Phoenix, and St. Louis). Candidate participants were screened based on the inclusion and exclusion criteria presented in Textbox 1. Participants were required to be adults who smoked cigarettes in the United States, aged between 21 and 64 years, who had smoked 100+ cigarettes in their lifetime and smoked cigarettes daily at the time of enrollment.

Textbox 1. **Inclusion and exclusion criteria for participation.**

**Inclusion criteria**

- Identify as male or female participant.
- Age 21 to 64 years old, current United States resident.
- Able to read and understand English.
- Own a qualifying smartphone for daily electronic diary entries.
- Smoked 100+ cigarettes in their lifetime.
- Currently smoke at least one cigarette daily.
- No intention to quit all tobacco and nicotine-containing products in the next 30 days.
- Report a positive overall opinion and a positive intention to try, use, and buy the study product (SP; after reviewing product information; responded good, very good, excellent when asked about overall opinion of the SP, and somewhat likely, very likely, or definitely when asked likelihood to try, use, and buy the SP).

**Exclusion criteria**

- No proof of age (eg, photo identification or driver’s license).
- Self-reported currently being pregnant or breastfeeding.
- Females of childbearing potential who were not using contraception.
- Employed in journalism, market research, public relations, in the tobacco industry, or as a health care provider.
- Participated in a consumer or clinical study on tobacco or nicotine-containing products in the past 30 days.
- Initiated cigarette or electronic nicotine delivery system use in the past 30 days.

Both exclusive cigarette smokers and dual users could benefit from a reduction in cigarette smoking; therefore, they were considered as separate study groups in this study. Qualifying adults who smoked cigarettes daily but did not use ENDS daily at the time of enrollment, nor had used ENDS 100+ times in their lifetime, were assigned as exclusive cigarette smokers. Qualifying adults who smoked cigarettes daily and used ENDS either daily or occasionally and had used ENDS 100+ times in their lifetime at the time of enrollment were enrolled as dual users. Participant enrollment and recruitment aimed to represent the sociodemographic characteristics of adults who smoke cigarettes daily in the United States, with marginal distributions of sex (male: 55%; female: 45%), age (21‐24 years: 10%; 25‐44 years: 50%; 45‐64 years: 40%), and race (White: 70%; non-White: 30%) based on data from the National Health Interview Survey (NHIS).

### Recruitment

The candidate participants were randomly selected and recruited from an external consumer database based on their smoking status and sociodemographic characteristics. Social media postings were also used to support recruitment. In a first step, candidate participants were screened against inclusion and exclusion criteria. Eligible participants were invited to the study site for rescreening and enrollment into the study. In a second step, eligible participants were invited to consent to being rescreened and exposed to the SP. Following exposure to the SP label, labeling, and marketing material, participants’ “Overall Opinion” and “Intention to Use” the SP were assessed. Participants who responded “good,” “very good,” or “excellent” on “Overall Opinion” and participants who responded “somewhat likely,” “very likely,” or “definitely” on “Intention to Try,” “Intention to Use,” and “Intention to Buy” were enrolled into the study to approximate product self-selection based on behavior intentions. This approach also protected participants by avoiding unnecessarily enrolling those who were not interested in the product.

Participants could voluntarily withdraw or be removed from the study if they did not report at least 4 electronic diary (e-diary) entries during the baseline period, failed to comply with study procedures, misused the SP, or became pregnant. All participants were compensated fairly for their time at the end of the observational period.

### Study Procedures

#### Study Products

The SP was the P4M3 Generation 2.0 System (Philip Morris Products S.A.) ENDS device with prefilled cartridges containing e-liquid (3.5%; 39 mg/mL nicotine) with 2 flavors: tobacco and menthol along with applicable safety warnings, labeling, and instructions for use. Participants received the SP at no cost from study sites during the observational period but had to pay for any other TNP they chose to use, which is an approach that has been used in previous tobacco use studies [33-37].

Participants obtained 10 cartridges at the beginning of the observational period and could select tobacco, menthol, or both variants; they were allowed to obtain additional cartridges during the study. A maximum number of SP cartridges available to each participant was applied based on the participant’s self-reported cigarette consumption at enrollment multiplied by a factor of 2. During the observational period, participants were repeatedly exposed to a reduced exposure claim:

SP produces significantly fewer and lower levels of harmful chemicals compared to cigarettes. Scientific studies have shown that switching completely from cigarettes to SP significantly reduces your body’s exposure to harmful chemicals. Important information: the SP is not risk-free.

The SP device and any unused cartridges had to be returned to the study sites at the end of the observational period. Each study site was responsible for maintaining records of the distribution and return of the SP during the study.

### Data Collection

Participants self-reported their daily use of cigarettes and other TNP (during the baseline and observational periods) and the SP, including the flavor variant used (during the observational period only) in an app-based e-diary. Participants were also interviewed 3 times during the observational period (following week 2, week 5, and week 8) to evaluate the SP’s sensory attributes (taste, smell, and aftertaste) and ease of use. Participants could report any potential adverse events or change in pregnancy status to a study-specific hotline number.

### Outcomes

Study outcomes included the following and are based on results at the end of the observational period (ie, week 8) versus baseline: (1) the proportion of participants who switched completely to the SP, (2) the absolute change in CPD, (3) the proportion of participants who reduced CPD by 50% or more, and (4) the proportion of participants who transitioned from daily to nondaily cigarette smoking. Each of the outcomes was assessed separately for exclusive cigarette smokers and dual users and was stratified by the SP variant(s) (only tobacco, only menthol, or both variants) used in week 8.

As continued SP use is associated with appealing product characteristics, the following were assessed at the end of the observational period: (1) ratings of liking of sensory attributes (taste, smell, and aftertaste) among users of the respective SP variant(s) and (2) ease of SP use [38].

### Measures and Definitions

All measures and definitions were based on those commonly used in national surveys on TNP use (eg, Population Assessment of Tobacco and Health [PATH]) [39] and from a similar actual use study [33]. At enrollment, participants were asked to report the following sociodemographic characteristics: age in years, sex, race, ethnicity, level of education, and annual household income.

At enrollment, current TNP use was assessed using the following categories: cigarettes, ENDS, moist snuff, chewing tobacco, snus, nicotine pouches, oral tobacco-free nicotine products, cigars, cigarillos, pipe tobacco, hookah or waterpipe tobacco (What is your current [insert TNP category] use behavior? [daily user, occasional user, or nonuser]). Participants also reported the following: current CPD (On average, how many cigarettes do you currently smoke per day? [numerical response]); flavor of cigarette (Is the brand of cigarettes you currently buy and smoke most often a menthol cigarette? [yes, no]); and intention to quit smoking (Are you seriously considering quitting smoking cigarettes in the next 6 months? [yes, no, don’t know]). Dual users were asked to report the flavor of e-liquid they used most often (What flavor of ENDS with nicotine do you currently buy and you, yourself, use most often?). The use of cigarettes and other TNP (as one overall category) was assessed each day during the baseline and observational periods, while SP and other ENDS were assessed each day during the observational period. Participants self-reported the total number of times they used the TNPs each day in an app-based e-diary. Daily and occasional use and the number of product uses per day were determined based on the number of entries for these products and were averaged across the specific week of the observational period. Change in average daily cigarette consumption was defined as the difference in average daily number of cigarettes consumed at week 8 compared to baseline.

Participants also reported the SP variant(s) they used daily. Switched completely to SP was defined as participants who reported using the SP but not smoking cigarettes or using other ENDS at week 8. Switched completely to ENDS was defined as participants who reported using the SP and other ENDS but not smoking cigarettes at week 8. Dual SP use was defined as participants who reported using the SP and cigarettes. Dual ENDS use was defined as participants who reported using the SP, other ENDS, and cigarettes at week 8.

Evaluation of SP sensory attributes (taste, smell, and aftertaste) and ease of use of SP were assessed via interviews. Each sensory attribute was evaluated using a 7-point Likert scale ranging from 1 (I don’t like it at all) to 7 (I like it very much). Ease of use of SP was assessed using a 7-point Likert scale ranging from 1 (not easy to use at all) to 7 (very easy to use).

### Sample Size Determination

The target sample sizes for exclusive cigarette smokers and dual users (study groups) were 500 and 375, respectively. Sample size calculations were based on an expected attrition rate of 40% for exclusive cigarette smokers and 20% for dual users, yielding a sample size of 300 participants in each study group at week 8 of the observational period. A higher attrition among exclusive cigarette smokers was expected, as they are less familiar with ENDS than dual users. The target sample sizes were also expected to provide a reliable estimation of the proportion of participants who switched completely to SP at week 8, with a minimum predefined level of precision of ±5% if the proportion was within the expected range of 5%‐20% for each study group.

### Data Analysis

Descriptive summary statistics were used to describe the study outcomes. Counts and percentages were calculated for categorical variables. Means, SDs, and 95% CIs were calculated for continuous and ordinal variables. Results were calculated for each outcome separately for exclusive cigarette smokers and dual users and stratified by the SP variant used at week 8. Results for each week of the observational period were based on the number of daily e-diary entries reported for each participant. The average product use was calculated if there were at least 4 daily e-diary entries that week; otherwise, data for that week were considered missing. Data analysis was conducted using SAS 9.4 (SAS Institute).

### Ethical Considerations

The study was approved by the Sterling Institutional Review Board (IRB ID: 9938). All participants provided written informed consent before participating in the study. The informed consent form included information that the findings may be published externally with no individual data disclosed. Participants were compensated US $100 for completing the on-site face-to-face screening interview, US $20 for completing each weekly e-diary assessment, US $35 for completing each follow-up phone interview, and an additional compensation for completing all study assessments and returning SPs was provided, with a maximum total compensation of US $500. The incentive of the on-site screening interview was increased by US $75 for exclusive cigarette smokers to ensure sufficient recruitment of this study group.

## Results

### Recruitment, Enrollment, and Participation

**Figure 2.**
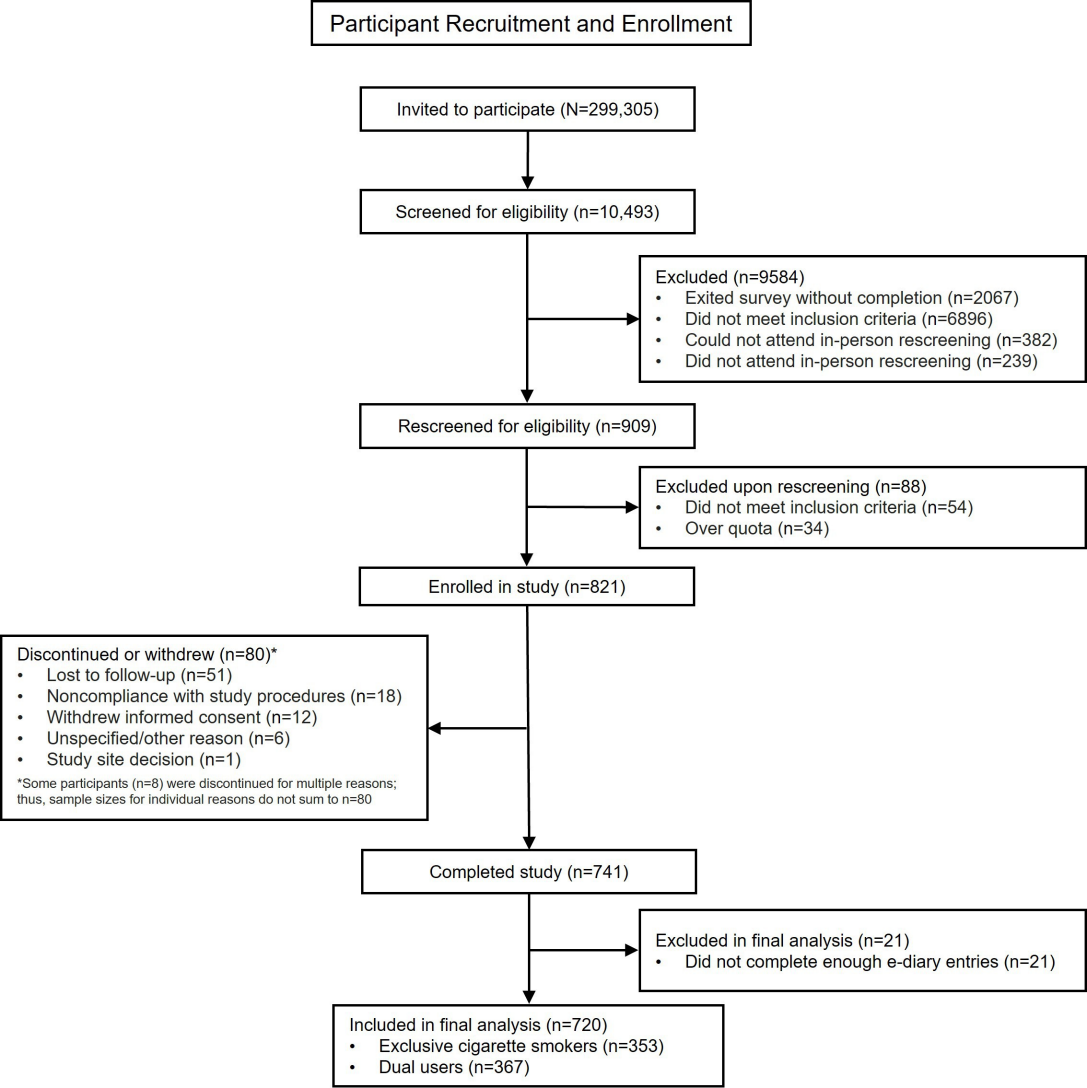
Participants’ recruitment and enrollment. e-diary: electronic diary.

There were 10,493 candidate participants initially screened for eligibility, with 909 qualifying and able to attend a study site for rescreening. Of the 909 rescreened qualifying participants, 821 were enrolled in the study (398 exclusive cigarette smokers; 423 dual users). There were 80 participants who discontinued or withdrew during the observational period, and 21 were excluded from the final analyses. Thus, 720 participants (353 exclusive cigarette smokers, attrition rate: 11.3%; 367 dual users, attrition rate: 13.2%) who completed the observational period and fulfilled the analysis criteria were included in the final analytical sample. Figure 2 details participants’ recruitment and enrollment.

### Sociodemographic Characteristics of the Study Participants

Exclusive cigarette smokers were slightly older than dual users (mean age 47, SD 10.4 years vs 44.3, SD 9.6 years) and contained fewer participants aged 21‐34 years old (45/353, 12.7% vs 63/367, 17.2%) and males (males: 126/353, 35.7% vs 181/367, 49.3%). Results for other sociodemographic characteristics are presented in Table 1.

**Table 1.** Sociodemographic characteristics of the study participants.

Sociodemographic characteristics	Exclusive cigarette smokers (n=353)	Dual users (n=367)
Age (years), mean (SD)	47 (10.4)	44.3 (9.6)
Age category (years), n (%)		
21‐24	6 (1.7)	5 (1.4)
25‐34	39 (11)	58 (15.8)
35‐44	100 (28.3)	122 (33.2)
45‐64	208 (58.9)	182 (49.6)
Sex, n (%)		
Male	126 (35.7)	181 (49.3)
Female	227 (64.3)	186 (50.7)
Ethnicity, n (%)		
Hispanic or Latino	54 (15.3)	37 (10.1)
Not Hispanic or Latino	299 (84.7)	330 (89.9)
Prefer not to answer	0 (0)	0 (0)
Race, n (%)		
White	226 (64)	228 (62.1)
Black	107 (30.3)	111 (30.2)
Another or multiple races	20 (5.7)	28 (7.6)
Prefer not to answer	0 (0)	0 (0)
Level of education, n (%)^a^		
Less than college graduate	242 (68.6)	197 (53.7)
College graduate or higher	111 (31.4)	170 (46.3)
Prefer not to answer	0 (0)	0 (0)
Annual household income, n (%)^b^		
Less than $75,000	217 (61.5)	205 (55.9)
$75,000 or higher	135 (38.2)	159 (43.3)
Prefer not to answer	0 (0)	2 (0.5)
Don’t know	1 (0.3)	1 (0.3)

a Level of education was based on the following response options: less than college graduate (less than high school; some high school or general education diploma; high school graduate; some college), college graduate or higher (college graduate; advanced degree), and prefer not to answer.

b Annual household income was based on the following response options: less than US $75,000 (less than US $10,000; US $10,000 to $29,999; US $30,000 to $44,999; US $45,000 to $59,999; US $60,000 to $74,999), US $75,000 or higher (US $75,000 to $99,999; US $100,000 to $149,999; US $150,000 or higher), prefer not to answer, and don’t know*.*

### Tobacco and Nicotine Product Use at Enrollment

Exclusive cigarette smokers and dual users smoked, on average, 13.3 (SD 7.3) and 12.1 (SD 7.2) CPD at enrollment, respectively. Nearly equal proportions reported they smoked menthol cigarettes (exclusive cigarette smokers: 197/353, 55.8%; dual users: 201/367, 54.8%). The proportion of exclusive cigarette smokers who had no plan to quit cigarette smoking in the next 6 months was 73.9% (261/353), while this proportion reached 89.4% (328/367) among dual users (Table 2).

**Table 2.** Tobacco and nicotine product use at enrollment.

Characteristics	Exclusive cigarette smokers (n=353)	Dual users (n=367)
Characteristics of cigarette smoking at enrollment		
Pattern of cigarette smoking, n (%)		
Adults who smoke daily	353 (100)	367 (100)
Frequency of cigarette smoking, mean (SD)		
CPD^a^	13.3 (7.3)	12.1 (7.2)
Type of cigarettes smoked, n (%)		
Menthol	197 (55.8)	201 (54.8)
Nonmenthol	156 (44.2)	166 (45.2)
Planned to quit smoking cigarettes in the next 6 months, n (%)		
Yes	54 (15.3)	34 (9.3)
No	261 (73.9)	328 (89.4)
Don’t know	38 (10.8)	5 (1.4)
Characteristics of other TNP^b^ use at enrollment		
Patterns of ENDS^c^ use, n (%)^d^		
Daily user	0 (0)	173 (47.1)
Occasional user	55 (15.6)	194 (52.9)
Nonuser	298 (84.4)	0 (0)
Flavor of ENDS used most often, n (%)^e^		
Tobacco	—	86 (23.4)
Menthol	—	146 (39.8)
Nontobacco and nonmenthol^f^	—	131 (35.7)
Some other flavor	—	4 (1.1)
Don’t know	—	0 (0)
Patterns of other TNP use, n (%)		
Moist snuff, chewing tobacco, or snus		
Daily user	1 (0.3)	13 (3.5)
Occasional user	16 (4.5)	72 (19.6)
Nonuser	336 (95.2)	282 (76.8)
Nicotine pouches		
Daily user	1 (0.3)	11 (3)
Occasional user	20 (5.7)	84 (22.9)
Nonuser	332 (94.1)	272 (74.1)
Cigars or cigarillos		
Daily user	23 (6.5)	49 (13.4)
Occasional user	118 (33.4)	171 (46.6)
Nonuser	212 (60.1)	147 (40.1)
Pipe tobacco		
Daily user	0 (0)	1 (0.3)
Occasional user	16 (4.5)	47 (12.8)
Nonuser	337 (95.5)	319 (86.9)
Hookah or waterpipe tobacco		
Daily user	0 (0)	10 (2.7)
Occasional user	87 (24.6)	153 (41.7)
Nonuser	266 (75.4)	204 (55.6)

a CPD: cigarettes per day.

b TNP: tobacco and nicotine-containing products.

c ENDS: electronic nicotine delivery systems.

d Adults who smoke cigarettes, who did not currently use electronic nicotine delivery systems daily nor had used them 100+ times in their lifetime, were enrolled in the exclusive cigarette smokers study group, whereas adults who smoke cigarettes, who currently used electronic nicotine delivery systems (daily or occasionally), and had used them 100+ times in their lifetime, were enrolled in the dual users study group.

e Exclusive cigarette smokers, who neither currently used electronic nicotine delivery systems daily nor had used them 100+ times in their lifetime, were not asked the flavor of electronic nicotine delivery systems they used most often.

f Nontobacco and nonmenthol electronic nicotine delivery systems flavor use included the following response options: fruit, chocolate, alcoholic or nonalcoholic drink, clove, spice, candy, desserts.

Among dual users, there were nearly equal proportions who were daily (173/367, 47.1%) or occasional (194/367, 52.9%) ENDS users. The majority of dual users reported using menthol (146/367, 39.8%) or tobacco (86/367, 23.4%) ENDS variants, while 36.8% (135/367) used other flavor variants. Information about other TNPs used at enrollment can be found in Table 2.

### Study Product Use and Assessment at Week 8

The e-diary compliance was high, with 96% (691/720) of participants in both study groups making 4 or more entries each week of the observational period.

At the end of the observational period (week 8), 86.2% (304/353) of exclusive cigarette smokers and 92.1% (338/367) of dual users used the SP. Dual users reported using the SP more often than exclusive cigarette smokers (mean 7.3, SD 12.3 times per day vs 5, SD 8.2 times per day). At week 8, 4% (14/353) of exclusive cigarette smokers and 4.1% (15/367) of dual users had switched completely to the SP. When considering the use of SP together with other ENDS, 4.5% (16/353) of exclusive cigarette smokers and 5.2% (19/367) of dual users had switched completely to ENDS at week 8. More participants in both study groups used only the menthol variant (exclusive cigarette smokers: 166/353, 54.6%; dual users: 167/367, 49.4%) compared to participants who used only the tobacco variant (exclusive cigarette smokers: 101/353, 33.2%; dual users: 112/367, 33.1%) and those who used both variants at week 8 (exclusive cigarette smokers: 37/353, 12.2%; dual users: 59/367, 17.5%).

The SP was favorably evaluated by participants in both study groups, with mean ratings for liking the sensory attributes ranging between 4.6 (SD 1.8) and 6.2 (SD 1.3) on a 7-point Likert scale. Notably, they liked the smell, taste, and aftertaste of the menthol variant on average more than the tobacco variant. Participants evaluated the SP as easy to use (mean 6.4, SD 1.2 vs 6.4, SD 1.1 on a 7-point Likert scale) (Table 3).

**Table 3.** Study product use and assessment at week 8.

Study product use	End of observational period (week 8)	
	Exclusive cigarette smokers (n=353)	Dual users (n=367)
SP^a^ use at week 8		
Patterns of SP use, n (%)^b^		
Used SP (any SP use)	304 (86.2)	338 (92.1)
Switched completely to SP (SP use without cigarettes)	14 (4)	15 (4.1)
Switched completely to ENDS^c^ (SP use and other ENDS without cigarettes)	2 (0.6)	4 (1.1)
Dual SP use (SP and cigarettes)	273 (77.3)	218 (59.4)
Dual ENDS use (SP and other ENDS and cigarettes)	15 (4.2)	101 (27.5)
Did not use SP (SP nonusers)	49 (13.8)	29 (7.9)
Frequency of SP use		
SP use, occasions per day		
Mean (SD)	5 (8.2)	7.3 (12.3)
95% CI^d^	4.1-5.9	6-8.5
Patterns of SP use by variant used^e^, n (%)		
SP variant used overall (n=304/n=338)		
Only SP tobacco variant	101 (33.2)	112 (33.1)
Only SP menthol variant	166 (54.6)	167 (49.4)
Both SP variants	37 (12.2)	59 (17.5)
Switched completely to SP by variant used (n=14/n=15)		
Only SP tobacco variant (n=101/n=112)	5 (5)	4 (3.6)
Only SP menthol variant (n=166/n=167)	8 (4.8)	8 (4.8)
Both SP variants (n=37/n=59)	1 (2.7)	3 (5.1)
SP flavor variants used by type of cigarettes smoked at enrollment, n (%)^f^		
Menthol cigarette smokers (n=174/n=187)		
Only SP tobacco variant	12 (6.9)	16 (8.6)
Only SP menthol variant	140 (80.5)	134 (71.7)
Both SP variants	22 (12.6)	37 (19.8)
Nonmenthol cigarette smokers (n=130/n=151)		
Only SP tobacco variant	89 (68.5)	96 (63.6)
Only SP menthol variant	26 (20)	33 (21.9)
Both SP variants	15 (11.5)	22 (14.6)
Assessment of SP used at final follow-up interview		
Liking of SP sensory attributes, mean (SD) [95% CI]^g^		
Taste		
Only SP tobacco variant (n=101/n=112)	4.8 (1.9), 4.5-5.2	4.6 (1.8), 4.3-5
Only SP menthol variant (n=166/n=167)	5.4 (1.5), 5.2-5.6	5.3 (1.5), 5.1-5.5
Both SP variants (n=37/n=59)	5.2 (1.4), 4.7-5.7	5.6 (1.3), 5.3-6
Smell		
Only SP tobacco variant (n=100/n=112)	5.9 (1.4), 5.6-6.1	5.5 (1.7), 5.2-5.9
Only SP menthol variant (n=162/n=163)	6.2 (1.3), 6-6.4	5.9 (1.4), 5.7-6.2
Both SP variants (n=37/n=59)	5.9 (1.4), 5.5-6.4	6.2 (1.2), 5.9-6.5
Aftertaste		
Only SP tobacco variant (n=100/n=112)	4.8 (1.9), 4.4-5.2	4.6 (1.9), 4.2-4.9
Only SP menthol variant (n=165/n=165)	5.3 (1.7), 5-5.6	5.2 (1.7), 4.9-5.4
Both SP variants (n=37/n=58)	5.2 (1.5), 4.8-5.7	5.4 (1.6), 4.9-5.8
Ease of use, mean (SD) [95% CI]^h^		
Ease of use	6.4 (1.2), 6.3-6.5	6.4 (1.1), 6.3-6.6

a SP: study product.

b Pattern of study product use definitions was based on use at week 8. Switched completely to study product (study product use without cigarettes) was defined as participants who reported using the study product but not smoking cigarettes or using other electronic nicotine delivery system at week 8. Switched completely to electronic nicotine delivery system (study product use and other electronic nicotine delivery system without cigarettes) was defined as participants who reported using the study product and other electronic nicotine delivery system but not smoking cigarettes at week 8. Dual study product use (study product and cigarettes) was defined as participants who reported using the study product and cigarettes. Dual electronic nicotine delivery system use (study product and other electronic nicotine delivery system and cigarettes) was defined as participants who reported using the study product, other electronic nicotine delivery system, and cigarettes at week 8.

c ENDS: electronic nicotine delivery systems.

d Calculated for participants who used the study product at week 8.

e Study product flavor variants used at week 8 were categorized as only tobacco SP variant, only menthol SP variant, or both SP variants.

f Study product nonusers are not included in these analyses.

g Liking of each sensory attribute was assessed using a 7-point Likert scale ranging from 1 (I don’t like it at all) to 7 (I like it very much); participants could also respond don’t know (not shown) among users of the respective SP variant(s) at week 8.

h Ease of use was assessed using a 7-point Likert scale ranging from 1 (Not easy to use at all) to 7 (Very easy to use); participants could also respond don’t know (not shown).

A total of 6 adverse events were reported over the course of the study. None were related to the use of the SP.

### Changes in Cigarette Consumption at Week 8

Participants in both study groups reduced their daily cigarette consumption from baseline to week 8 by 3.1 CPD among exclusive cigarette smokers and by 2.5 CPD among dual users. About one-quarter of exclusive cigarette smokers (91/353, 25.8%) and dual users (103/367, 28.1%) reduced CPD by 50% or more (Table 4).

**Table 4.** Changes in cigarette smoking from baseline to week 8.

Changes in cigarette smoking	Exclusive cigarette smokers (n=353)		Dual users (n=367)	
	Baseline	Week 8	Baseline	Week 8
Characteristics of cigarette smoking at week 8				
Frequency of cigarette smoking, mean (SD), [95% CI]^a^				
Average CPD^b^	12.4 (7.1),11.6-13.1	9.3 (7.5), 8.5-10.1	10.7 (7.2), 10-11.5	8.3 (7.3), 7.5-9
Absolute change from baseline	—^c^	–3.1 (4.5), –3.5 to 2.6	*—*	–2.5 (4.4), –2.9 to –2
Days without cigarette smoking among people who smoke daily				
n	6	64	30	98
Days per week, mean (SD), 95% CI	1.5 (0.8), 0.6-2.4	3.8 (2.4), 3.2-4.4	2.2 (1.5), 1.6-2.8	3.6 (2.3), 3.2-4.1
CPD reduction >50% from baseline, n (%)				
Reduced CPD >50%	—	91 (25.8)	—	103 (28.1)
Cigarette smoking by SP^d^ variant used at week 8^e^				
Patterns of cigarette smoking by SP variant used, n (%)^f^				
Only SP tobacco variant (n=101/n=112)				
Daily user	101 (100)	86 (85.1)	104 (92.9)	92 (82.1)
Nondaily user	0 (0)	10 (9.9)	8 (7.1)	15 (13.4)
Nonuser	—	5 (5)	—	5 (4.5)
Only SP menthol variant (n=166/n=167)				
Daily smoker	163 (98.2)	135 (81.3)	151 (90.4)	124 (74.3)
Nondaily smoker	3 (1.8)	25 (15.1)	16 (9.6)	35 (21)
Nonsmoker	—	6 (3.6)	—	8 (4.8)
Both SP variants (n=37/n=59)				
Daily smoker	37 (100)	27 (73)	56 (94.9)	34 (57.6)
Nondaily smoker	0 (0)	9 (24.3)	3 (5.1)	20 (33.9)
Nonsmoker	—	1 (2.7)	—	5 (8.5)
Frequency of cigarette use (CPD) by SP variant used, mean (SD), 95% CI^a^				
Average CPD				
Only SP tobacco variant (n=101/n=112)	13.4 (7.5), 12-14.9	10 (6.8), 8.7-11.4	12 (7.2), 10.7-13.4	9.7 (7.4), 8.3-11.1
Absolute change from baseline	—	–3.4 (4.7), –4.3 to –2.5	—	–2.3 (4.8), –3.2 to –1.4
Only SP menthol variant (n=166/n=167)	12.1 (6.8), 11.1-13.1	8.7 (7.3), 7.6-9.9	10.4 (7.4), 9.3-11.5	7.7 (6.8), 6.7-8.8
Absolute change from baseline	—	–3.4 (4.6), –4.1 to –2.6	—	–2.7 (4.4), –3.4 to –2
Both SP variants (n=37/n=59)	10.5 (5.8), 8.6-12.4	7.5 (6.9), 5.2-9.8	8.2 (5.2), 6.8-9.6	5.6 (5.5), 4.1-7
Absolute change from baseline	—	–3 (3.8), –4.3 to –1.8	—	–2.6 (3.6), –3.5 to –1.7
CPD reduction>50% from baseline by SP variant used, n (%)				
Reduced CPD>50%				
Only SP tobacco variant (n=101/N=112)	—	18 (17.8)	—	24 (21.4)
Only SP menthol variant (n=166/N=167)	—	52 (31.3)	—	48 (28.7)
Both SP variants (n=37/N=59)	—	11 (29.7)	—	23 (39)
Patterns of cigarette smoking, n (%)^g^				
Daily smoker	347 (98.3)	289 (81.9)	337 (91.8)	269 (73.3)
Nondaily smoker	6 (1.7)	49 (13.9)	30 (8.2)	77 (21)
Nonsmoker	—	15 (4.2)	—	21 (5.7)

a CPD calculated only among participants who smoked cigarettes at week 8.

b CPD: cigarettes per day.

c Not available.

d SP: study product.

e SP flavor variants used at week 8 were categorized as only tobacco SP variant, only menthol SP variant, or both SP variants.

f Results for daily and nondaily smoking when stratified by SP flavor variants used may not equal the results for daily and nondaily smoking overall given some participants did not use the SP at week 8.

g Daily cigarette smoking was an inclusion criterion for study enrollment and all (100%) participants in both study groups reported daily cigarette smoking. Results for the use pattern of cigarette smoking were based on data at baseline, during which some exclusive cigarette smokers (n=6) and dual users (n=30) did not smoke daily.

The proportion of participants who reported smoking less than daily increased from baseline to week 8 among exclusive cigarette smokers (from 1.7% (6/353) to 13.9% (49/353) and dual users (from 8.2% (30/367) to 21% (77/367)).

### Study Results by SP Variant Used at the End of the Observational Period

The majority of cigarette smokers who used menthol and nonmenthol cigarettes at enrollment used the congruent flavor SP variant at week 8. However, higher proportions of nonmenthol cigarette smokers used the menthol SP variant than menthol cigarette smokers who used the tobacco SP variant (Table 3).

At the end of the observational study, the proportions of participants who switched completely to the SP were 5% (5/101) and 3.6% (4/112) among exclusive cigarette smokers and dual users, respectively, among those who used only the tobacco SP variant, while this proportion was similar in both study groups among those who used only the menthol SP variant (8/166, 4.8% and 8/167, 4.8%). The proportion of participants who switched completely to SP was 2.7% (1/37) among exclusive cigarette smokers and 5.1% (3/59) among dual users among those who used both SP variants (Table 3).

Regarding changes in cigarette smoking, the reductions in CPD were similar by flavor SP variant(s) used, ranging from 3 CPD among participants who used both SP variants to 3.4 CPD among participants who used only the tobacco SP variant or who used only the menthol SP variant among exclusive cigarette smokers. Among dual users, the reduction in CPD was 2.3 CPD among those who used only the tobacco SP variant, 2.6 CPD among those who used both SP variants, and 2.7 CPD among those who used only the menthol SP variant. The proportions of exclusive cigarette smokers and dual users who reduced CPD by 50% or more were higher among those who used both SP variants (11/37, 29.7% and 23/59, 39%, respectively) or only the menthol SP variant (52/166, 31.3% and 48/167, 28.7%, respectively) versus only the tobacco SP variant (18/101, 17.8% and 24/112, 21.4%, respectively) (Table 4).

The proportion of participants who reported smoking less than daily increased, from baseline to week 8, the most among participants who used both SP variants (exclusive cigarette smokers: 0% to 9/37, 24.3%; dual users: 3/59, 5.1% to 20/59, 33.9%), followed by those who used only the menthol SP variant (exclusive cigarette smokers: 3/166, 1.8% to 25/166, 15.1%; dual users: 16/167, 9.6% to 35/167, 21%), then by those who used only the tobacco SP variant (exclusive cigarette smokers: 0 to 10/101, 9.9%; dual users: 8/112, 7.1% to 15/112, 13.4%) (Table 4).

## Discussion

Adults who smoke cigarettes and completely switch to ENDS or substantially reduce cigarette consumption are exposed to far lower levels of inhaled toxicants [40] and report positive changes in their health compared to adults who smoke cigarettes and continue to smoke cigarettes [41]. Hence, if a sufficient number of adults who smoke cigarettes switch to these products, it is likely to be beneficial to population health [7].

This study examined the use patterns of a novel ENDS among adults who smoke cigarettes with no or limited experience using ENDS (exclusive cigarette smokers) and those using other ENDS (dual users). These tobacco users are the intended users of the SP, as both could benefit from switching completely from cigarette smoking [1]. Results showed high acceptance of the SP, as 86.2% (304/353) of exclusive cigarette smokers and 92.1% (338/367) of dual users used the SP at the end of the observational period (week 8). Some exclusive cigarette smokers (14/353, 4%) and dual users (15/367, 4.1%) switched completely from cigarettes to the SP, and over a quarter of all participants reduced CPD by >50% (91/353, 25.8% of exclusive cigarette smokers and 103/367, 28.1% of dual users) by week 8. The levels of reduction at week 8 among all participants equated to smoking about 3 fewer CPD, corresponding to over one fewer pack of cigarettes per week (20 cigarettes/pack) than at baseline.

The findings of this study need to be considered in the context of other studies assessing the use of ENDS among adults who smoke cigarettes in near real-world conditions. In line with our study results, Carpenter et al [42] found that, at the end of a 4-week period during which participants received a free supply of a commercially available ENDS, approximately 70% of adults who smoke cigarettes adopted the ENDS product, 10% completely switched to ENDS, and 33% reduced their CPD by >50% compared to baseline. A recent actual use study on ENDS among adults who smoke cigarettes showed that after a 1-week trial period and a 6-week use period, past-7-day cigarette abstinence ranged from 38.2% to 47.3% across 5 flavor variants and between 50.9% to 62.9% of AS who continued smoking reduced their daily cigarette consumption by at least 50% compared to baseline [43,44]. The superior outcomes observed in this study are likely attributable to the availability of a wider range of flavor variants as well as to differences in study design, specifically the inclusion of a product trial period after enrollment to identify participants with a positive interest in using and purchasing the SP before commencing the 6-week actual use period with their preferred flavor variant.

This study further supports existing evidence that e-cigarettes can help smokers to replace or substantially reduce cigarette consumption. Cross-sectional studies on tobacco use transitions among adults in the United States have found that exclusive current ENDS users are more likely to be former cigarette smokers and that dual users smoked fewer cigarettes after initiating ENDS [45,46]. Similar findings have been shown in other countries [47-49]. Cohort studies on tobacco use demonstrate that adults who smoke cigarettes and use ENDS experience a reduction in CPD, transition from daily to nondaily smoking, have increased attempts to, and successfully quit smoking, with stronger evidence for daily versus nondaily ENDS use [50-61]. Results from a recent systematic review of randomized controlled trials provide evidence of the increased efficacy of ENDS compared to conventional methods of quitting smoking, including NRT, behavioral support, and no intervention [11].

Findings of other studies showed that sensory attributes, nicotine concentration, and device type influence ENDS liking and use [62-71]. In this study, both study groups liked the taste, smell, and aftertaste of both SP variants, but with greater average liking for the menthol variant. This finding is likely to explain both the higher use proportions and the higher substantial reductions in cigarette consumption among those who used the SP menthol variant in both study groups at week 8. The higher liking and use prevalence of the menthol ENDS variant measured in this study have also been reported in previous studies [44,65,72,73], while Selya et al [44] also found that the use of menthol ENDS variants increases the likelihood of complete switching.

This study had several strengths. The study design was similar to previous actual use studies [33-35]. Adults who smoke cigarettes were allowed to use the SP ad libitum in near real-world conditions, allowing participants to establish their pattern of use over an extended period (8 weeks). Participants were recruited from 7 geographically diverse metropolitan areas in the United States, and the sample approximated that of the United States adult population who smoke cigarettes with respect to sex, age, and race. Finally, the study provided estimates for 2 populations: exclusive cigarette smokers and dual users, who might benefit from switching completely from cigarette smoking.

The study was not without limitations. Participants did not have to pay for the SP, which differs from the real-world conditions, but did have to pay for their cigarettes and other TNP. This might have affected their preference for the SP, increasing complete switching to SP and reducing cigarette consumption. The SP was available in 2 variants (tobacco and menthol) and at one nicotine concentration (3.5%; 39 mg/mL nicotine), which might have limited its adoption and use among dual users who may be familiar with a larger variety of flavor variants and nicotine concentration of ENDS [65]. Participants were compensated fairly for participation, but the impact on TNP use during the study was likely limited as payment was provided after study completion. Use of SP and all other TNP was self-reported and may be subject to recall bias, though self-reported TNP use is shown to reliably measure actual TNP use [74].

### Conclusions

Results of this actual use study show that exclusive cigarette smokers and dual users of cigarettes and ENDS found the SP appealing, leading some of them to switch completely to the SP or substantially reduce their cigarette consumption. Both study groups liked the sensory experience of the tobacco and menthol variants and found the SP easy to use. These results complement existing scientific evidence that ENDS are an acceptable alternative to cigarettes for adults who smoke in the United States.

## Abbreviations

CPD: cigarettes per day
e-diary: electronic diary
ENDS: electronic nicotine delivery system
NHIS: National Health Interview Survey
NRT: nicotine replacement therapies
PATH: Population Assessment of Tobacco and Health
SP: study product
TNP: tobacco and nicotine-containing products
